# Developmental Origins of Non-Communicable Chronic Diseases: Role of Fetal Undernutrition and Gut Dysbiosis in Infancy

**DOI:** 10.3390/children11111387

**Published:** 2024-11-15

**Authors:** Manju Chandra

**Affiliations:** Division of Pediatric Nephrology, Department of Pediatrics, New York University Grossman Long Island School of Medicine, Mineola, NY 11501, USA; manju.chandra@nyulangone.org

**Keywords:** DOHaD, NCCD, IUGR, gut microbiome

## Abstract

There is an increasing prevalence of non-communicable chronic diseases (NCCDs) like obesity, metabolic syndrome, type 2 diabetes mellitus (T2DM), hypertension, allergic asthma, and neuro-developmental/psychiatric problems in many parts of the world. A suboptimal lifestyle as an adult is often blamed for the occurrence of NCCDs. This review discusses the developmental origin of health and disease theory and how suboptimal nutrition in intrauterine life and the establishment of a suboptimal gut microbiome during infancy can influence the predisposition to NCCDs.

## 1. Introduction

There is an increasing prevalence of non-communicable chronic diseases (NCCDs) like obesity, metabolic syndrome, type 2 diabetes mellitus (T2DM), hypertension, allergic asthma, and neuro-developmental/psychiatric problems in many parts of the world, making NCCDs a leading cause of disability, morbidity, and mortality [1,2]. Some of these NCCDs are presenting at a younger age, especially in socioeconomically disadvantaged communities. NCCDs are often attributed to unhealthy personal lifestyles. However, there is evidence to suggest that certain environmental exposures during early life can predispose the development of NCCDs in adulthood. The developmental origins of health and disease (DOHaD) theory proposes that future health is influenced by environmental exposures during the critical windows of early life, including in utero, infancy, and early childhood [3,4,5].

Most early studies supporting the DOHaD theory focused on the effects of fetal undernutrition and intrauterine growth restriction (IUGR) on the subsequent development of obesity, hypertension, and cardiovascular disease [3,4], but there is increasing interest in evaluating the effects of other early life environmental exposures like the gut microbiome, since early interventions for preventing NCCDs can then be examined and implemented. Studies show that the establishment of a rich and diverse symbiotic gut microbiome in the first three years of life promotes health, while a loss of diversity and abundance of beneficial symbiotic gut microbes can predispose the development of the aforementioned NCCDs [6,7,8,9]. Studies show that environmental exposures during the period of developmental plasticity can mediate metabolic changes and an altered phenotype due to changes in gene expression.

This review is intended to increase the awareness of DOHaD and the importance of the gut microbiome and is based on a literature search for articles published in the fields of DOHaD, IUGR, the gut microbiome, and their association with NCCDs.

## 2. Relationship of NCCDs to Intrauterine Growth Restriction

Individuals born with IUGR due to an inadequate nutrient supply in utero have been noted to have a higher risk of certain health problems later in life, including, but not limited to, hypertension, proteinuria, obesity, T2DM, cardiovascular disease, and neurodevelopmental and psychiatric problems such as impulsive behavior, anxiety, and depression [5,10,11,12,13,14,15,16,17,18]. The constellation of these problems is a result of developmental conditioning and the altered metabolic programming of the fetus occurring in order not only to survive in utero under nutrient deprivation but also to survive ex utero in an anticipated similar nutritionally challenged environment.

The fetal metabolic changes in response to undernutrition have been well elucidated in previous reports [5] and include strategies to decrease growth rate and energy demand in fetal life and to prepare the fetus for energy scarcity ex utero. Some of these include the development of insulin and leptin resistance and the redistribution of blood flow by vascular remodeling to allow relatively more nutrition to reach the heart, lungs, brain, and adrenals, while rationing nutrition distribution to certain other developing organ systems [19,20].

The adaptations in response to undernutrition in fetal life lead to predisposition for NCCDs in several ways. Leptin resistance results in decreased energy expenditure, increased capacity for fat storage, lack of satiety, and a hyperalert state to forage for food, all of which predispose the development of obesity and metabolic syndrome when the extrauterine environment affords plenty of nutrition, and rapid catch up growth occurs in the first year of life. Impaired glucose metabolism from insulin resistance and less pancreatic beta cell endowment from reduced blood flow to the developing pancreas predispose the development of T2DM [13,17,18]. Nutritional rationing to the kidneys results in lower nephron endowment, increased workload per nephron, and nephron damage, with resultant proteinuria and hypertension [21]. IUGR further predisposes the development of hypertension for other reasons, including a smaller diameter of the aorta, a decreased distensibility of certain arteries due to reduced elastin vs. collagen content in the vessel wall, and from increased salt sensitivity [19,20,22,23]. Fetuses with IUGR have increased exposure to maternal cortisol from a decrease in the activity of the placental enzyme 11 beta—hydroxysteroid2, which converts maternal cortisol to its inactive form, cortisone. Increased fetal exposure to cortisol alters the hypothalamic–pituitary–adrenal axis, thereby affecting neurodevelopment and subsequently increasing the risk of anxiety, attention deficit hyperactivity disorder, and psychiatric problems [5,12,24].

A long-term human study was conducted on the offspring of mothers who were exposed to 6 months of famine during pregnancy in the Dutch Hunger Winter of 1944–1945 during World War II [25]. It was noted that the offspring of mothers exposed to undernutrition in the first and second trimesters exhibited a higher risk of obesity, hypertension, cardiovascular disease, and T2DM in adult life, but the offspring exposed to caloric deprivation in the third trimester did not display these same risks, underscoring the importance of the developmental programming of metabolism in early fetal life [25].

IUGR, which is often associated with premature birth, is likely to become an increasingly prevalent cause of NCCDs in some parts of the world. In the USA, the incidence of premature birth (at less than 37 weeks of gestation) among live births is approximately 10%, with IUGR noted in 5%, low birth weight of less than 2500 g in 8.5%, and very low birth weight of less than 1500 g in 1.4% of these cases [21]. With advances in neonatal care, the majority of the low birth weight, as well as many very low birth weight infants now survive into adulthood and may develop NCCDs. Perinatal history and a related higher risk of NCCDs in this population should be highlighted in their medical records in order to provide appropriate screening and management.

## 3. Epigenetic Responses and DOHaD

Early life nutrition and environmental exposures can affect phenotype and disease risk later in life through epigenetic modifications of gene expression [24]. The transcription of the genes is known to be suppressed or enhanced by cytosine methylation/demethylation within the CpG dinucleotides of DNA and histone modification by deacetylation/acetylation. Furthermore, interfering microRNAs can inhibit the translation of the transcribed mRNA into protein.

Most epigenetic and metabolic programming resulting from dietary and environmental factors occurs during the period of high plasticity of the organism when it can most respond to change. Generally, for humans, this crucial time consists of the first 1000 days of life, including the 280 days of gestation and the 730 days comprising the first 2 years of life, as well as during gametogenesis. Environmental influences on the epigenome are influenced by both the dose and the developmental timing [26]. Stressors during pregnancy have been linked to the subsequent development of hypertension and obesity in offspring [5,27], and stressors in early life have been shown to result in poor growth and behavioral problems.

Studies in Agouti viable yellow mice shed light on the impact of altered nutrition during pregnancy on modifying disease risk [28]. Agouti VY mice have a mutant Agouti gene which is constantly turned on. Compared to wild type mice, which express brown coats, Agouti viable yellow mice display a yellow coat, a ravenous appetite, become very obese early in life, and have a propensity for developing T2DMs and cancer. Mothers with the mutant Agouti gene give birth to pups with a range of coat colors, from brown to yellow. However, when the yellow mothers were fed a diet rich in genistein, a phytoestrogen from soybean, starting just before conception and throughout pregnancy, the mothers’ diet resulted in changes in the phenotype of the offspring. Most of the offspring of these yellow mothers then displayed a brown coat color, and they did not develop obesity, T2DM, or cancer [28]. The change in phenotype of the litter resulted from the increased methylation of the CpG sites in their Agouti gene, inhibiting its expression. The extent of this DNA methylation was similar in the endodermal, mesodermal, and ectodermal tissues, indicating that genistein acts during early embryonic development. Moreover, this genistein-induced hypermethylation persisted into adulthood, decreasing ectopic Agouti gene expression and protecting the offspring from obesity. This study provided the first evidence that in utero dietary manipulation affects gene expression and alters susceptibility to obesity in adulthood by permanently altering the epigenome [28].

In another study, Agouti viable yellow mice exposed to bisphenol, (a chemical often present in plastics) produced litters with more pups with yellow coats. However, when the mice fed bisphenol were also given dietary supplements including methyl-donor foods [29], such as choline, folic acid, vitamin B12, and betaine, the phenotype changed, and there were more offspring of brown coat color. While bisphenol reduces methylation, a diet rich in methyl donors was able to overcome the effects of bisphenol and suppress the expression of the Agouti gene.

The life course of the honeybee exemplifies an alteration in phenotype resulting from a change in early life nutrition. While the honeybee queen and worker bees have the same DNA sequence, the females are phenotypically and temperamentally quite different. The differential phenotype between the queen and the worker bees is due to the epigenetic effects of differential nutrition between the larvae [30,31]. While all developing larvae are fed royal jelly for the first 3 days of life, those few chosen to be raised as queens continue to be fed royal jelly after this timepoint, while the other larvae are fed honey only. The differential diet provides substrates for epigenetic writers and erasers, resulting in the suppression or activation of certain genes in the larvae, producing a differential phenotype.

## 4. Transgenerational Epigenetic Inheritance

The altered epigenome can result in the transgenerational inheritance of health or disease risk, since it may be transmitted to the offspring [32,33,34].

Stewart et al. undernourished rats with a protein deficient diet over 12 generations [35]. When re-fed with a normal diet, it took three generations before fetal growth and development were restored to normal. This study suggests that epigenetic change is an adaptive process which responds to the environment; it allows genotypic variation to be preserved through transient environmental changes.

Transgenerational epigenetic inheritance can contribute to social determinants of health, since some families and communities may be repeatedly exposed to nutritional deprivation or environmental toxins in the workplace or at home [32,33].

## 5. Composition and Relevance of Gut Microbiome

The environmental factors which can result in altered epigenetic and metabolic programming are not always external to the human body but may be chemicals produced by the microbial flora on and in the human body [7]. Humans are host to trillions of microbes that live in and on our bodies. Diverse families of microbes, including bacteria, fungi, and viruses, live on human skin and mucosal surfaces in a rich ecosystem. About 95% of human microbiota live in the gut, and most of them reside in the colon. Humans have 10 times more microbial cells than human cells and have over 3 million microbial genes. The census data of the microbial population is generally evaluated by microbial signatures in the stool, obtained through shotgun metagenomics and the amplification of 16 S ribosomal RNA sequence analysis. The microbiome is the collective genomic information contained within the microbiota, and the word is used to describe microbial characteristics and effects.

Healthy gut microbiota mainly comprise two phyla, Firmicutes and Bacteroidetes, which represent 90% of gut microbiota, but it also contain less-represented phyla, such as Actinobacteria [36,37,38]. The phylum Firmicutes includes several genera, of which the majority are Lactobacillus, Bacillus, Enterococcus, Ruminicoccus, and Clostridium. Bacteroidetes consists of predominant genera such as Bacteroides and Prevotella. The Actinobacteria phylum is mainly represented by the Bifidobacterium genus. The microbes in the colon thrive on undigested food fibers and resistant starches, fermenting them to produce metabolites which affect human metabolism.

While humans are more complex and intelligent living creatures than many on earth, they have only 23,000 functioning genes, as compared to about 14,000 genes in fruit flies and 46,000 genes in some rice plants [39]. The complexity of human functions is partly dependent on the work outsourced to the microbes. The human gut microbiome serves as the “second genome”. The microbes constantly sense the environment and communicate with the rest of the human body via their metabolites; the latter serve as substrates for the activities of the epigenetic modification enzymes and influence the expression of host genes. Since the microbes have a short lifespan of hours and reproduce rapidly compared to humans, their microbiome can more rapidly and effectively induce metabolic changes in the human body than can the human genome in response to environmental exposures.

## 6. Establishment of a Symbiotic and Diverse Microbiome in Infancy

Despite the vast variety and numbers of microbes found in the adult gut, the microbiota of the infant gut is initially a simple ecosystem which gradually undergoes successional changes until it reaches high diversity. The development of the infant gut microbiota is profoundly influenced by the host genotype; gestational age; mode of delivery; diet; antibiotic use; exposure to siblings, pets, and a rural vs. urban environment [40,41,42,43,44]. At birth, the founder/pioneer species of the microbiome are acquired from the mother’s birth canal and from skin to skin contact [40,45]. Breast feeding promotes colonization by symbiotic microbes. Human milk is comprised of 15% human milk oligonucleotides, which do not have caloric value but which selectively promote the growth of beneficial microbes, like Bifidobacterium, in the gut. As infants grow, they continue to acquire diverse microbes from a more varied diet of fruits, vegetables, and fermented foods and from contact with other people, pets, livestock, soil, and things they touch [41]. The founder species help dictate the subsequent acquisition of complementary microbes.

The diversity and abundance of a beneficial microbial community expands the most during the first year of life but slows down after about age 3. By then, people develop a distinct microbiome which reflects a specific combination of microbial species due to their environmental exposures and dietary habits [40,41,42,43,44,46]. The diversity and abundance of the gut microbiome is crucial to the stability of the microbial population, since a rich and biodiverse ecosystem is resistant to change and can bounce back if acutely disturbed by antibiotic therapy or a high dose of acquired pathogens [47]. Diet seems to be one of the most important influences on the acquisition of a stable symbiotic microbiome [41]. Colonic microbes thrive on undigested plant fibers, fermenting them. Because of the structural discrepancies between different types of fiber, many different enzymes are needed to break it down, and not every species of bacteria produce all the enzymes necessary to break down all types of fiber. Different species of bacteria work together as commensal organisms, with varying capabilities to hydrolyze different dietary fibers; therefore, eating a variety of high-fiber foods such as fruits, vegetables, whole grains, and legumes can encourage greater microbial diversity.

The metabolites of the rapidly diversifying microbiome in infancy influence the gut barrier function and motility; regulate caloric expenditure, satiety, and energy balance; affect the development of the brain and neural connections; and train the naïve immune system to recognize friends and foes to prevent allergies and autoimmune diseases. A disruption in the acquisition of a stable and diverse microbiome in the first 3 years of life can have lasting adverse effects.

## 7. Influence of Infant Gut Microbiome on Future Health and Disease

The following are some of the ways that human gut microbes and their metabolites affect health. Symbiotic resident microbes prevent gut infections through competitive growth inhibition of pathogenic microbes. Colonic microbes produce short-chain fatty acids (SCFAs) like butyrate, propionate, and acetate via the fermentation of undigested plant fibers and carbohydrates. SCFAs nourish the gut mucosa and promote the integrity of the gut barrier function by increasing the density of the tight junctions, increasing mucus production, and inducing both the differentiation and apoptosis of colonic cells [48,49]. The integrity of the intestinal barrier is essential for health, since increased intestinal permeability results in the translocation of microbial products into systemic circulation, which can promote systemic inflammation and insulin resistance. SCFAs produced in the colon also enter the blood stream and affect remote organs, where they prevent inflammation, increase glucagon like peptide 1 production, improve glucose homeostasis, and increase satiety, thus preventing obesity and the development of T2DM [50,51,52,53].

Microbial products influence the release and function of gut hormones which affect gut motility, perception of visceral pain, appetite, glucose metabolism, and insulin secretion. Alterations in gut microbiota composition can dysregulate the enteroendocrine signaling pathways, contributing to irritable bowel syndrome, as well as the metabolic disturbances associated with diabetes mellitus [50,54,55].

There is bidirectional communication between the enteric nervous system and the central nervous system. In infants, gut microbiome metabolites influence the CNS development, affecting the maturation of microglia, the myelination process, and blood–brain barrier function. The gut microbes produce various neurotransmitters which affect behavior and mental health, e.g., serotonin produced from dietary tryptophan promotes happiness and satisfaction, dopamine affects the reward centers of the brain and mechanisms for coping with stress, while melatonin production helps promote sleep [56,57,58,59]. Altered gut microbiota have been associated with altered neurodevelopment and neuropsychiatric problems [53,59,60]. On the other hand, early life toxic stress can adversely affect the quality of the gut microbiome and thereby further affect mental health, while also causing a predisposition for hypertension [26,27].

The gut hosts 80% of the body’s immune cells in its Peyer’s patches, and through their dendrites, the immune cells are able to sense intestinal luminal chemicals and learn which microbes and foods are to be tolerated or rejected [6,61,62,63]. The GI microbiome guides the maturation of the naive infant immune system to antigen exposure and primes the immune system toward tolerogenic T cells, as compared to the proallergic/pro-inflammatory T cell response. The training of the naïve immune system occurs in the first 3 years of life and confers susceptibility or resistance to allergic or autoimmune diseases [63].

GI microbiome metabolites influence the risk of obesity via effects on energy harvesting from undigested carbohydrates and fibers; caloric expenditure; and the regulation of appetite, food choices, and food cravings [55,59]. A person’s lifestyle can affect the composition of the microbiome and the risk of obesity and hypertension, as well as metabolic health [19,55,64,65,66],since the gut microbiota exhibit diurnal oscillation that can be influenced by feeding rhythms and the synchrony of the sleep–wake cycle with sunlight and exercise frequency [67,68].

The loss of compositional diversity and the abundance of beneficial symbiotic organisms in the gut, changes in their local distribution and metabolic activities, and excessive growth of potentially harmful organisms are labeled as “dysbiosis”. The occurrence of dysbiosis in the first 3 years of life has been associated with an increased risk for obesity, T2DM, hypertension, allergic and autoimmune diseases, and neurodevelopmental and neuropsychiatric problems. A less diverse and depleted microbiome caused by antibiotic usage in both livestock and human infants has been shown to promote later weight gain [69,70,71]. In both humans and mice, the recipients of fecal microbiota transplant from obese or lean microbiomes exhibit the phenotypes of the donors [72,73].

## 8. Increasing Prevalence of Dysbiosis in Industrialized Countries

Recent studies suggest an increasing prevalence of dysbiosis in industrialized countries [69]. Several factors have contributed to the increased dysbiosis in industrialized nations vs. less prosperous countries. There are higher rates of cesarian section rather than natural vaginal delivery [42,43]; a higher prevalence of formula feeding over breastfeeding; higher antibiotic usage during pregnancy and infancy; the use of food production and preservation practices which result in unintended exposure to antibiotics, pesticides, and food additives; diets low in plant fiber; less food variety; and less intake of local seasonal produce. There is also less contact with the natural world, since the majority of time at work or at home is spent in an indoor sanitized environment with controlled temperature, humidity, and filtered air, thus decreasing the exposure to local seasonal microbes. Dysbiosis is also promoted by the disruption of the normal circadian rhythm-induced physiologic metabolic processes due to the asynchrony of the sleep–wake cycle with the sun and prolonged exposure to blue light from electronic devices in the evening [67,74].

## 9. Best Practices to Establish and Maintain the Optimal Microbiome in Infancy and Beyond

In order to obtain long term health, a healthy and diverse gut microbiome needs to be established in infancy and maintained in the long term. The traditional, thousands of years old sayings attributed to Indian Ayurveda medicine and Hippocrates that “all diseases begin in the gut” are corroborated by modern studies showing the importance of the microbiome.

The best practices to establish an optimal microbiome start with the pregnant mother, e.g., diet, healthy lifestyle, vaginal delivery, breast feeding for the first 6 months, and avoidance of unintentional exposure to antibiotics and pesticides. The process continues in infancy and childhood, with the intake of diverse foods high in plant fiber, which serve as prebiotics; the intake of fermented foods, which serve as probiotics; plenty of physical activity; and optimal environmental exposures to local fauna and flora, while avoiding or minimizing exposures to toxins, pesticides, and antibiotics. Time-restricted eating in adults, with a 12-h period overnight with no food, is recommended to optimize health [75]. Proper sleep hygiene and exercise should be promoted. While some stress is unavoidable, habitual implementation of strategies to cope with stress, such as participation in rich social interactions and non-competitive group play, should be encouraged.

Potential therapeutic approaches to improve the gut microbiome in those with dysbiosis include supplements of probiotics and postbiotics, as well as fecal microbiota transplantation [76,77]. In patients with recurrent clostridium difficile infection, cure rates of 90–95% have been achieved with microbiome modulation from fecal microbiota transplantation [78].

## 10. Conclusions

Adverse environmental influences in early life, such as poor nutrition, exposures to antibiotics or certain chemicals, toxic stress, and dysbiosis, can influence the predisposition for NCCDs later in life. This occurs from altered metabolic and epigenetic programming during the period of high developmental plasticity when a person can most respond to change. For humans, generally, this crucial time occurs during the first 1000 days of life, including the 280 days of gestation and first 2 years of life (730 days), as well as during gametogenesis. The socioeconomic disparities in health trajectories may in part be mediated by the effects of adverse events in early life and transgenerational epigenetic inheritance. Any intervention to prevent and treat NCCDs, such as the promotion of healthy eating habits and lifestyle, is best initiated during the period of developmental plasticity to produce the maximal effect. The establishment of a rich and diverse symbiotic microbiome in infancy can promote long-term health and may decrease the risk for some NCCDs.
